# Supplementary material for: Heavy Metals in Sediment from the Urban and Rural Rivers in Harbin City, Northeast China
Source: Int J Environ Res Public Health. 2019 Nov 6;16(22):4313. doi: 10.3390/ijerph16224313 (PMC6888195; doi:10.3390/ijerph16224313)
Supplement: Supplementary file 1 [file ijerph-16-04313-s001.pdf]

International Journal of Environmental Research and Public Health

MDPI

Article

Heavy Metals in Sediment from the Urban and Rural Rivers in Harbin City, Northeast China

Song Cui [1,*], Fuxiang Zhang [1], Peng Hu [2], Rupert Hough [3], Qiang Fu [1], Zulin Zhang [3], Lihui An [4], Yi-Fan Li [5], Kunyang Li [1], Dong Liu [1] and Pengyu Chen [1]

Table S1. Classifications of heavy metal pollution degree and potential ecological risk.

P_i	P_N		Pollution Degree	E_r^i	RI	Ecological Risk
	Traditional	Improved				
$P_i \le 1$	$P_N \le 1$	$P_N' \le 1$	Clean	$E_r^i < 40$	$RI < 55$	Low
$1 < P_i \le 2$	$1 < P_N \le 2.5$	$1 < P_N \le 2.6$	Low	$40 \le E_r^i < 80$	$55 \le RI < 110$	Moderate
$2 < P_i \le 3$	$2.5 < P_N \le 7$	$2.6 < P_N \le 6.4$	Moderate	$80 \le E_r^i \le 160$	$110 \le RI < 220$	High
$P_i > 3$	$P_N > 7$	$P_N > 6.4$	High	$160 \le E_r^i < 320$	$RI \ge 220$	Very high
				$E_r^i \ge 320$		Serious

Table S2. Comparison of heavy metal concentrations in surface sediments.

Location		Cd (mg/kg)	Cu (mg/kg)	Cr (mg/kg)	Zn (mg/kg)	Pb (mg/kg)	Ni (mg/kg)	Reference
Majiagou River, China	Range	0.08–4.1	4.0–82.5	75.1–203.2	128.2–1416.7	8.9–57.5	7.9–30.4	This study
	Mean	0.8	28.1	107.4	358.5	27.0	17.8	
Yunliang River, China	Range	BDL–4.3	15.8–22.3	53.7–81.9	113.2–2474.1	9.3–114.4	BDL–13.1	This study
	Mean	1.8	19.5	68.2	861.6	32.8	8.2	
Xiangjiang River, China	Range	4.25–31.2	24.6–250.1	67.9–170.0	30.7–1009.7	25.5–672.3	16.0–187.2	[1]
	Mean	13.7 **	101.4 **	120.4 **	443.3	214.9 **	57.1 **	
Xiangjiang River, China	Range	1.69–15.64	18.6–78.4	31.9–88.9	71.5–397.3	23.8–104.7	—	[2]
	Mean	6.8 **	45.2 **	52.7 **	221.6	66.1 **	—	
Yangtze River, China	Range	0.06–0.3	11.7–46.6	10.5–113.0	44.5–125.0	14.8–32.7	—	[3]
	Mean	0.2 *	28.0	52.1 **	77.6 *	21.9	—	
Yellow River, China	Range	BDL–0.25	7.0–261.0	42.6–132.0	41.8–114.0	4.3–42.5	15.0–39.6	[4]
	Mean	0.1 *	40.7 **	62.4 **	68.4 **	15.2 *	23.6 **	
Songhua River, China	Mean	0.3	13.3*	121.4 **	92.5 *	18.8	12.9	[5]
Louro River, Spain	Range	0.37–1.4	30.5–55.9	78.1–139.0	—	43.6–91.1	32.5–60.7	[6]
	Mean	0.7	45.4 **	108.0 **	—	61.8 **	46.4 **	
Gorges River, Australia	Range	—	2.0–138.0	3.0–126.0	7.0–788.0	3.0–267.0	0.7–38.0	[7]
	Mean	—	30.0	39.0 **	157.0 *	67.0 **	13.0	
Gironde Estuary, France	Range	0.01–2.1	0.5–40.1	1.3–140.0	4.0–323.0	5.0–83.8	0.9–48.4	[8]
	Mean	0.5	24.5	78.4	168.0 *	46.8 **	31.7 **	

Note: BDL below detection limit; ** Difference is significant at $p < 0.01$ level (two-tailed); * Difference is significant at $p < 0.05$ level (two-tailed).

Table S3. Pearson correlation matrix for heavy metals in surface sediments of the Yunliang River.

	Cu	Cr	Zn	Pb	Ni	Cd
Cu	1					
Cr	0.582	1				
Zn	−0.143	0.112	1			
Pb	−0.771	−0.501	0.224	1		
Ni	0.298	0.668	0.343	−0.606	1	
Cd	−0.182	0.129	0.997**	.225	0.376	1

** Correlation is significant at $p< 0.01$ level (two-tailed); * Correlation is significant at $p< 0.05$ level (two-tailed).

Table S4. Pearson correlation matrix for heavy metals in surface sediments of the Majiagou River.

	Cu	Cr	Zn	Pb	Ni	Cd
Cu	1					
Cr	0.454	1				
Zn	0.125	0.102	1			
Pb	0.539	0.164	0.794 **	1		
Ni	0.401	0.741 **	0.529	0.53	1	
Cd	0.157	0.369	0.950 **	0.727 **	0.677 *	1

** Correlation is significant at $p< 0.01$ level (two-tailed); * Correlation is significant at $p< 0.05$ level (two-tailed).

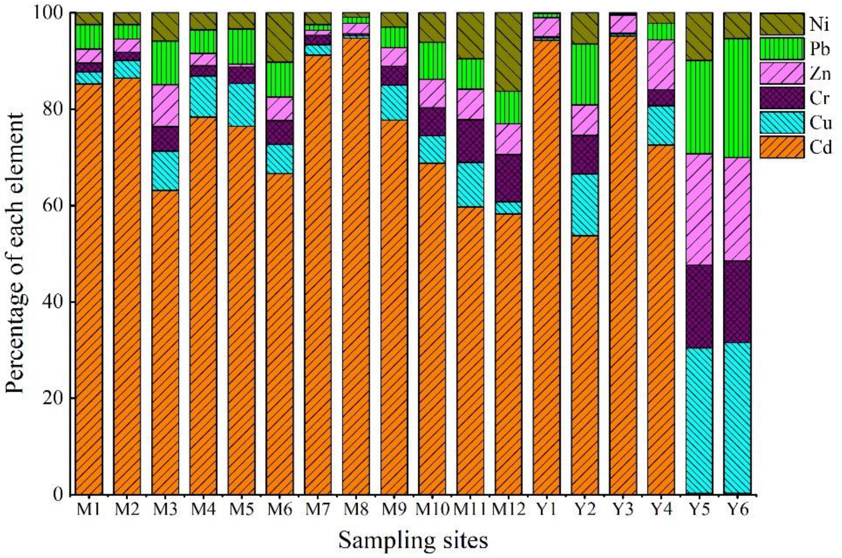

Figure S1. Contribution of different heavy metals to the ecological risk index (*RI*) in riverine sediments.

References

1. Chai, L.; Li, H.; Yang, Z.; Min, X.; Liao, Q.; Liu, Y.; Men, S.; Yan, Y.; Xu, J. Heavy Metals and Metalloids in the Surface Sediments of the Xiangjiang River, Hunan, China: Distribution, Contamination, and Ecological Risk Assessment. *Environ. Sci. Pollut. Res.* **2016**, *24*, 874–885.
2. Li., D.; Pi, J.; Zhang, T.; Tan, X.; Fraser, D. Evaluating a 5-Year Metal Contamination Remediation and the Biomonitoring Potential of a Freshwater Gastropod along the Xiangjiang River, China. *Environ. Sci. Pollut. Res.* **2018**, *25*, 21127–21137.
3. An, Q.; Wu, Y.; Wang, J.; Li, Z. Heavy Metals and Polychlorinated Biphenyls in Sediments of the Yangtze River Estuary, China. *Environ. Earth Sci.* **2009**, *59*, 363–370.
4. Yan, N.; Liu, W.; Xie, H.; Gao, L.; Han, Y.; Wang, M.; Li, H. Distribution and Assessment of Heavy Metals in the Surface Sediment of Yellow River, China. *J. Environ. Sci.* **2016**, *39*, 45–51.
5. Li, N.; Tian, Y.; Zhang, J.; Zuo, W.; Zhan, W.; Zhang, J. Heavy Metal Contamination Status and Source Apportionment in Sediments of Songhua River Harbin Region, Northeast China. *Environ. Sci. Pollut. Res.* **2017**, *24*, 3214–3225.
6. Filgueiras, A. V.; Lavilla, I.; Bendicho, C. Evaluation of distribution, mobility and binding behaviour of heavy metals in surficial sediments of Louro River (galicia, spain) using chemometric analysis: a case study. *Sci. Total Environ*. **2004**, *330*, 115–129.
7. Alyazichi, Y. M.; Jones, B. G.; Mclean, E. J.; Pease, J.; Brown, H. K. Geochemical assessment of trace element pollution in surface sediments from the Georges River, Southern Sydney, Australia. *Arch. Environ. Contam. Toxicol.* **2016**, *72*, 1–13.
8. Larrose, A.; Coynel, A.; SchaFer, J.; Blanc, G.; Masse, L.; Maneux, E. Assessing the Current State of the Gironde Estuary by Mapping Priority Contaminant Distribution and Risk Potential in Surface Sediment. *Appl. Geochem*. **2010**, *25*, 1912–1923.

© 2019 by the authors. Licensee MDPI, Basel, Switzerland. This article is an open access article distributed under the terms and conditions of the Creative Commons Attribution (CC BY) license (http://creativecommons.org/licenses/by/4.0/).
